# Combination therapy for patent ductus arteriosus in preterm infants: Narrative review

**DOI:** 10.1177/19345798251337433

**Published:** 2025-05-07

**Authors:** Eyad Bitar, Aimann Surak, Kumar Kumaran, Abbas Hyderi

**Affiliations:** 1 Queen’s University, Kingston, ON, Canada; 2 3158 University of Alberta, Edmonton, AB, Canada

**Keywords:** Acetaminophen, combination therapy, nonsteroidal anti-inflammatory drugs, patent ductus arteriosus, preterm infants

## Abstract

Management of patent ductus arteriosus (PDA) in preterm infants remains controversial and is a matter of continuous debate with a lack of consensus among practitioners on the optimal therapeutic strategy. The success rate of the most commonly used agents for PDA closure is variable, despite different medications, dosage regimens, routes of administration, and timing or duration of treatment. Combination therapy works by inhibiting prostaglandin production at different levels on the synthesis pathway; so combining acetaminophen and nonsteroidal anti-inflammatory drugs might potentially enhance PDA closure. Few studies explored the effectiveness and safety profile of combination therapy. This review summarizes the best available evidence on the efficacy and safety profile of combination pharmacological therapy for PDA treatment in preterm infants.

## Introduction

The ductus arteriosus (DA) plays a crucial role as a vascular shunt during fetal life, facilitating communication between the pulmonary and systemic circulations. During intrauterine development, the majority of the right ventricular output traverses the duct, bypassing the pulmonary circulation.^1^ Following birth, the DA undergoes a functional closure, and subsequently a permanent closure within a few days among term infants.^2^ When the DA remains patent beyond that period, a patent ductus arteriosus (PDA) is diagnosed, which is the case in most extremely preterm infants.^3^

Various factors modulate the patency of the duct, with imbalance favoring its patency in preterm infants. For instance, 87% of infants born at 24 weeks gestational age (GA) exhibit a PDA at 7 days of life.^4^ When hemodynamically significant, PDA is associated with significant morbidity and mortality, particularly among extremely preterm infants born at <28 weeks GA or with a birth weight (BW) <1000 grams (g).^5^ The optimal approach for managing PDA in extremely preterm infants is a matter of debate.^6^ Additionally, the efficacy of pharmacological therapeutic strategies is suboptimal, with closure rates for commonly used agents such as indomethacin, ibuprofen, and acetaminophen ranging from 45% to 76% after a single course.^7,8^ In addition, repeated courses are often needed to achieve higher closure rates.^9^ Given the current success rates of different therapeutic strategies and protocols, an optimal and safe approach is yet to be established. This may involve modifications to dosing regimens, routes of administration, or the selection of medical therapy. Combination therapy for PDA closure has been proposed; however, there is a paucity of evidence in the literature about this strategy. This approach recognizes that the process of PDA closure is complex and multifactorial, often involving multiple mechanisms. By sequentially blocking or modulating these pathways, clinicians aim to optimize treatment efficacy while minimizing adverse effects.

## Pharmacological management

During fetal development, DA remains open primarily due to the influence of prostaglandins (PGs), particularly Prostaglandin E2 (PGE2), synthesized from membranous phospholipids.^10^ PGE2 promotes cyclic adenosine monophosphate (cAMP) formation, maintaining vasodilation, while nitric oxide (NO) stimulates cyclic guanosine monophosphate (cGMP) production, further inducing vasodilation.^11^ In utero patency of the DA is critical, as its closure is associated with severe fetal complications.^12^ Postnatally, physiological changes such as decreased pulmonary vascular resistance and reduced PGE2 production contribute to the closure of DA.^13^ Preterm infants face challenges in DA closure due to their physiological immaturity and increased sensitivity to prostaglandins and nitric oxide, leading to potential delays or failure in closure.^14,15^

**Figure 1.**
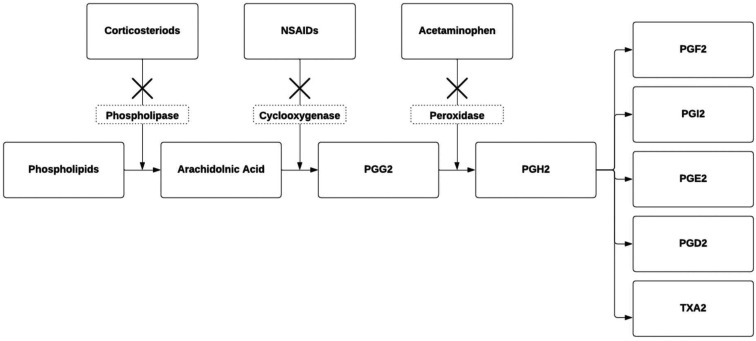
Prostaglandin synthesis pathway. NSAIDs, non-steroidal anti-inflammatory drugs; PG, prostaglandin; TX, thromboxane.

The available pharmacological agents employed for inducing ductal closure operate through the prostaglandin synthesis pathway. Non-steroidal anti-inflammatory drugs (NSAIDs) inhibit the cyclooxygenase pathway, while acetaminophen works on the peroxidase pathway (Figure 1). Several factors, such as gestational age and race, influence the ductal response to treatment, rendering PDA in preterm infants more resistant to constricting drug therapy.^16^

NSAIDs work by reducing the production of PGs by blocking the conversion of arachidonic acid into PGG2 (Figure 1). There are a few NSAIDs that are commonly used in the pediatric and neonatal populations including indomethacin, ibuprofen, and aspirin.

### Monotherapy

#### Indomethacin

Since it was first described for its role in closing PDA in 1976, indomethacin has been widely used with evidence-based effectiveness in therapeutic use.^17^ However, a meta-analysis of the prophylactic use of indomethacin for PDA in preterm infants reported only short-term benefits (decreased risk of severe intraventricular hemorrhage and less need for surgical ligation), with no long-term neurodevelopmental benefits.^18^ There are different dosage regimens ranging from 0.1 mg/kg to 0.3 mg/kg per dose every 12 to 24 h with variable effectiveness.^17^ No significant benefit was found for extending its use.^19^ Indomethacin use is associated with several potential side effects, mainly related to vasoconstrictive effects on cerebral, renal, and mesenteric vascular beds.^20,21^ Although the vasoconstriction is transient, this could potentially be complicated with necrotizing enterocolitis (NEC) and bowel perforation.^21,22^

#### Ibuprofen

Ibuprofen is another NSAID agent that has been used widely for PDA closure in the last 2 decades. Ibuprofen is reported to be as effective as indomethacin in closing the PDA, with fewer side effects.^23^ Typically, a standard ibuprofen regimen includes a loading dose of 10 mg/kg followed by 5 mg/kg administered twice every 12–24 h. This can be administered orally or intravenously. A high-dose course (15–20 mg/kg loading, followed by 2 more doses of 7.5–10 mg/kg every 12–24 h) has been proposed more effective without increasing the rate of adverse effects.^24^ Ibuprofen may cause oliguria, gastrointestinal hemorrhage, and possibly elevated pulmonary vascular resistance.^25^

#### Acetaminophen

For the last decade, acetaminophen has gained more popularity for the treatment of PDA in preterm infants. It was found that it is as effective as NSAIDs with a favorable safety profile. The proposed mechanism of action of acetaminophen is based on the inhibition of PGH2 (Figure 1). In clinical trials, acetaminophen showed similar efficacy to indomethacin and ibuprofen with a lower incidence of kidney injury, thrombocytopenia, gastrointestinal bleeding, and hyperbilirubinemia.^26^ Acetaminophen is usually given at 15 mg/kg/dose 4 times a day for 3–7 days orally or intravenously.^27,28^

#### Aspirin

In addition, maternal aspirin use has been shown to decrease the incidence of hemodynamically significant patent ductus arteriosus (hsPDA) in preterm infants and to increase the response to postnatal medical therapy.^29^ Only one trial evaluated the use of aspirin for ductal closure in preterm infants and found it less effective in comparison to indomethacin.^30^ The partial inhibition of cyclooxygenase, as well as potential salicylate toxicity, made aspirin out of favor in the neonatal population.

### Combination pharmacological therapy

Combination therapy using more than one agent has drawn attention.^31^ It is speculated that combining acetaminophen and an NSAID agent could inhibit PGE-2 production more effectively by the different mechanistic actions of PGH-2 and PGG-2, respectively (Figure 1). This has been used as an effective treatment of fever in infants and children with no additional adverse effects.^32^

Researchers at Vanderbilt University Medical Center investigated how ibuprofen and acetaminophen affect ductal tissue using a mouse model. They used pressure myography to assess isolated ductus arteriosus segments from late gestation mice. Findings revealed that the combination of ibuprofen and acetaminophen led to notably higher ductus arteriosus constriction compared to using either drug alone. This suggests a possible synergistic effect, highlighting a potential combined benefit of these medications on ductal tissue reactivity.^33^

Few human studies explored the effectiveness and safety of combination pharmacological therapy in the management of PDA in preterm neonates. Table 1 represents a summary of these studies.

**Table 1.** Summary of the included studies.

Study	Design	Size	Population	Time	HsPDA criteria	Intervention	Control	Closure rate intervention vs control			
								After 1^st^ course	*p*-Value	After 2^nd^ course	*p*-Value
Hochwald et al. (2018)	RCT	24	Mean GA = 27.5 (1.4) weeks	Mean= 6.5 ± 1.4 days	Clinical & Echo	(*n* = 12) IV acetaminophen 20 mg/kg Q6h X 3 days and IV ibuprofen 10 mg/kg then 5 mg/kg X 2	(*n* = 12) IV ibuprofen 10 mg/kg then 5 mg/kg X 2	50% vs 25%	0.4	83% vs 42%	0.08
Yurttutan et al. (2019)	Prospective case series	12	Median GA = 28 (26–36) weeks	Median = 22.5 (15–32) days	Clinical & Echo	PO acetaminophen 15 mg/kg Q6h X 5 days and PO ibuprofen 10 mg/kg then 5 mg/kg X 2	—	75% vs NA	NA	NA	—
Oboodi et al. (2020)	RCT	154	Mean GA = 31 (2.6) weeks	Mean = 5.5 ± 3.9 days	Clinical & Echo	(*n* = 19) IV acetaminophen 20 mg/kg Q6h X 3 days and PO ibuprofen 10 mg/kg then 5 mg/kg X 2	(*n* = 135) IV acetaminophen 20 mg/kg Q6h X 3 days or PO ibuprofen 10 mg/kg then 5 mg/kg X 2	78.9% vs 76.3%	0.97	100% vs 88.9%	0.41
Kimani et al. (2020)	Retrospective cohort	140	Median GA = 25.7–26.1 weeks	Median = 6.5–7 days	Echo	(*n* = 17) PO acetaminophen 15 mg/kg Q6h X 7 days and IV or PO ibuprofen 10 mg/kg then 5 mg/kg X 2	(*n* = 123) IV indomethacin 0.2 mg/kg/dose X 3 days or PO acetaminophen 15 mg/kg Q6h X 7 days or IV or PO ibuprofen 10 mg/kg then 5 mg/kg X 2	41.2% vs 39%	0.1	NA	—
Aikaio et al. (2020)	Retrospective cohort	121	Median GA = 25.7–26.1 weeks	Median = 3.0 (1–6) days	NA	(*n* = 18) IV acetaminophen 20 mg/kg then 7.5 mg/kg q6h and PO ibuprofen 10 mg/kg then 5 mg/kg X 2	(*n* = 103) PO ibuprofen 10 mg/kg then 5 mg/kg X 2	61.1 % vs 46.6%	0.26	NA	—
Shah et al. (2021)	Prospective case–control	31	Mean GA = 25.2 (1.3) weeks	Mean = 26.5 ± 1.4 cGA	Clinical	(*n* = 11) PO acetaminophen 15 mg/kg Q6h X 3 days and PO ibuprofen 10 mg/kg then 5 mg/kg X 2	(*n* = 20) IV ibuprofen 10 mg/kg then 5 mg/kg X 2	55% vs 36%	0.46	NA	—
Saker et al. (2023)	Retrospective cohort	32	Mean GA = 25.8 (1.1) weeks	Median = 10 days	Echo	(*n* = 32) PO/IV/PR acetaminophen 15 mg/kg Q6h X 7 days and PO/IV ibuprofen 10 mg/kg then 5 mg/kg X 2	—	42.2%	—	NA	—

GA, corrected gestational age; HsPDA, hemodynamically significant patent ductus; IV, intravenously; RCT, randomized controlled trial; PO, orally.

Hochwald et al. conducted a pilot RCT of 24 preterm infants 24 to 31^6/7^ weeks GA to test the effect of combination therapy on hsPDA.^34^ Infants in the study arm received IV ibuprofen + IV acetaminophen, while infants in the control arm received IV ibuprofen + IV placebo (normal saline 0.9%). The echocardiographic markers included a duct diameter >1.5 mm and at least one of the following: a left atrium-to-aorta ratio (LA:Ao) >1.5, unrestrictive pulsatile ductal flow, and end-diastolic reversal blood flow across the descending aorta. Up to 2 courses of the same treatment were offered. Mean GAs for both treatment and control groups were comparable (Table 1). The closure rates after 2 courses were 83% (combination therapy) versus 42% (control), *p*-value = 0.08 with no difference for any of the secondary outcomes (need for a second course, need for surgical ligation, and rates of ductal reopening). Renal and liver function tests were not different between the 2 groups. Despite a small sample size, the trial showed a trend toward higher closure rates after repeated courses of combination therapy compared to monotherapy.

A case series by Yurttutan et al. reported the effect of combination therapy with oral acetaminophen and ibuprofen on 12 preterm infants (26–36 weeks GA) who failed to respond to 2 courses of monotherapy.^35^ The regimen included a 5-day course of acetaminophen and 3 doses of ibuprofen (Table 1). Ductal closure occurred in 9 of the 12 patients (75%), and authors suggested a possible beneficial effect of dual therapy over monotherapy for resistant PDA before considering ligation. However, the absence of a control group limits the findings of this study.

Oboodi et al. randomized 154 preterm infants <37 weeks GA with hsPDA into 3 groups: the acetaminophen group (*n* = 67), ibuprofen group (*n* = 68), and combination therapy group (acetaminophen + ibuprofen) (*n* = 19).^36^ Infants with a clinical suspicion of a duct in the first 14 days of life underwent echocardiography to identify those with hsPDA. Acetaminophen IV was given at 15 mg/kg every 6 h for 3 days, and oral ibuprofen at the initial dose of 10 mg/kg followed by 5 mg/kg every 24 h (for a total of 3 doses). After the first course, the closure rate was not different among the 3 groups (76.1%, 76.4%, and 78.9%, respectively; *p* = 0.97). However, after a second course of treatment, the closure rate approached 100% in the combination group, compared to 86.6% in the acetaminophen group and 91.2% in the ibuprofen group (*p*-value = 0.41). There were no significant differences in the pre- and post-treatment laboratory values for the 3 treatment modalities, other than mild thrombocytosis seen in the combination medical therapy arm. Similar to Hochwald et al.,^34^ this trial showed that this approach was safe (no evidence of hematologic, hepatic, and renal impairment). In addition, the single dual therapy course was not superior to monotherapy.

Kimani et al.,^37^ retrospectively analyzed a cohort of 140 infants born <32 weeks GA. Seventeen infants received combination therapy of oral acetaminophen and ibuprofen (IV or PO), 29 received oral acetaminophen, 22 infants had ibuprofen (IV or PO), and 72 had IV indomethacin (Table 1). The echocardiographic evidence of hsPDA was defined as follows: PDA diameter >1.5 mm, unrestrictive flow pattern, LA/Ao > 1.6; diastolic turbulence in the main pulmonary artery and evidence of ductal steal. There was no statistically significant difference in the ductal closure among the 4 treatment groups (combination therapy [41.2%], indomethacin [41.7%], acetaminophen [37.9%], and ibuprofen [31.8%], *p*-value = 0.1). Combination therapy did not increase the risk for nephrotoxicity, hepatotoxicity, spontaneous intestinal perforation, NEC, or severe thrombocytopenia.

Another retrospective review by Aikio et al.,^38^ investigated whether the simultaneous administration of acetaminophen for pain therapy enhances the closure of the ductus arteriosus. The researchers collected data from infants with a GA <32 weeks and/or a BW <1500 g, diagnosed with hsPDA, and treated with ibuprofen. The data from 18 infants who received both acetaminophen and ibuprofen and 103 infants who received only ibuprofen were analyzed. The main finding of the study was that the infants who received combination therapy required fewer repeat PDA treatments than those who received only ibuprofen. Specifically, 5 out of 18 infants in the combination therapy group required repeat treatment, while 45 out of 103 infants in the ibuprofen-only group required repeat treatment. This difference was found to be statistically significant. The study concluded that simultaneous administration of acetaminophen may enhance the closure of the PDA in very low gestational age infants. However, further randomized trials are necessary to confirm the efficacy and safety of this potential treatment option.

In a case–control study, Shah et al. evaluated the efficacy of combination therapy with PO ibuprofen and acetaminophen for the closure of hsPDA in infants born <29 weeks GA and BW <1000 g at ≤14 postnatal days.^39^ Combination therapy was administered to 20 infants which was compared to 11 infants who received single medical therapy with IV ibuprofen. The dosing regimen included PO acetaminophen at 15 mg/kg every 6 h for 3 days along with PO ibuprofen at an initial dose of 10 mg/kg followed by 2 additional 5 mg/kg doses 24 and 48 h after the initial dose. The primary outcome was the rate of ductal closure. Secondary outcomes included repeated medical treatment, rate of ductal reopening, PDA ligation, echocardiographic measurement of the duct, mortality, and several different morbidities (hematologic, hepatic, and renal). In this study, dual therapy did decrease the ductal size. However, when compared to monotherapy, dual therapy did not show more success in closing or decreasing the size of the duct (11/20 [55%] vs 4/11 [36%], *p* = 0.46; Table 1). Also, the study reported no harm associated with combination therapy. There was no difference in pretreatment and up to 5 days of posttreatment in the levels of aspartate aminotransferase, alanine aminotransferase, serum bilirubin, and creatinine for the dual therapy group. There was also no difference in serum creatinine levels between the 2 groups during the 3 days of study treatment. A major limitation was the small sample size, in addition to the study design (nonrandomized case–control). Ibuprofen IV did not result in a statistically significant reduction in PDA size (mean difference = 0.20 mm, 95% CI: −0.13 to 0.65, adjusted *p*-value = 0.414), which does not correlate with the established published evidence and could be related to the small sample size in the control group (number = 11 infants). Finally, the study attempted to compare PO combination therapy to IV ibuprofen, and as known previously, oral ibuprofen is more effective than the IV form in achieving ductal closure.^24^ Therefore, any possible difference between the two groups in this study could be related to the mode of administration (PO vs IV).

A retrospective cohort study by Saker et al. evaluated the efficacy and safety of combining ibuprofen and acetaminophen for treating hsPDA in preterm infants.^40^ Notably, the study aimed to assess changes in echocardiographic parameters before and after combination therapy. Results revealed an overall treatment success rate of 41%, with a notably higher success rate observed when combination therapy was utilized as a primary course (55%) rather than as rescue therapy (17%). The echocardiographic analysis demonstrated significant improvements in hemodynamic parameters with primary combination therapy, suggesting its potential as an effective first-line treatment option. However, further research is warranted to explore the safety and efficacy of this approach in broader clinical settings.

Finally, there are 2 ongoing registered RCTs to evaluate the clinical impact, efficacy, and safety of combination treatment regimes. ACEDUCT Trial (NCT05340582) is a multicenter, double-blinded, placebo-controlled study that aims to evaluate the combination of acetaminophen and ibuprofen against monotherapy with ibuprofen for the first course of treatment in premature infants <27 weeks GA with unrestricted PDA ≥1.5 mm. The primary outcome is a composite of pre-discharge mortality or any grade bronchopulmonary dysplasia. The study is aiming to recruit 310 infants with estimated completion in 2026. A pilot study (NCT03648437) is also investigating the effectiveness and safety of administering simultaneous medications containing ibuprofen or indomethacin and paracetamol for the closure of PDA in preterm infants less than 37 weeks and is expected to complete recruitment in 2024.

**Figure 2.**
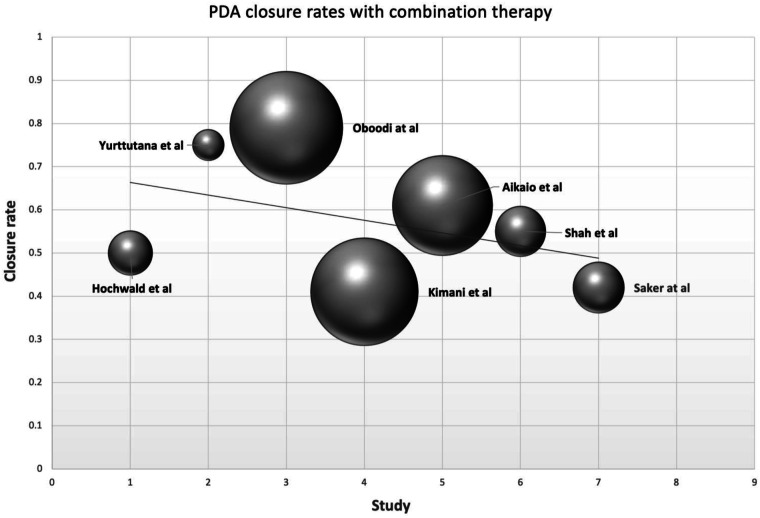
Bubble plot for PDA closure rates with combination therapy.

**Figure 3.**
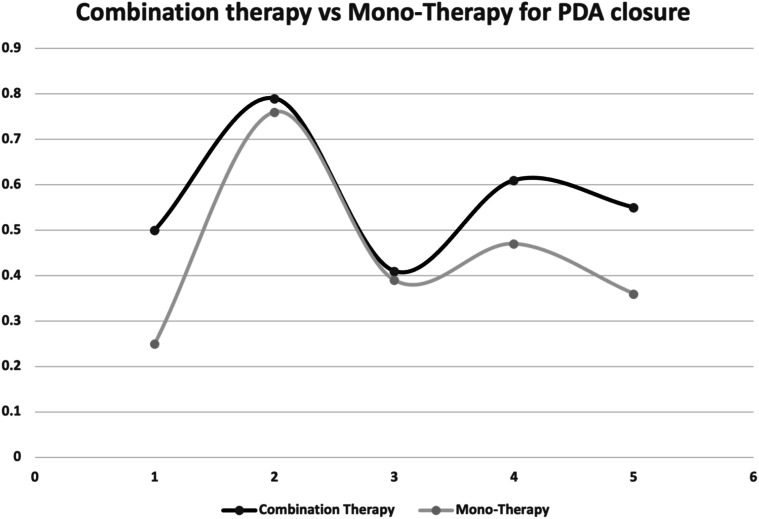
Comparison of PDA closure rates between combination therapy and monotherapy.

Figure 2 visualizes the bubble plot of PDA closure rates across the different studies with combination therapy. Each bubble represents a study, with the size of the bubble corresponding to the sample size of the study. A comparison between the closure rates for combination therapy and mono-therapy is represented in Figure 3.

## Discussion

The management of PDA in preterm infants typically utilizing NSAIDs or alternative agents like acetaminophen has long been considered the standard approach. These medications aim to achieve ductal closure by inhibiting PG synthesis, thus interrupting the patency of the DA. However, a notable proportion of preterm infants fail to respond adequately to monotherapy, leading to persistent or recurrent PDA and necessitating alternative strategies for management. In response, researchers have explored the potential benefits of combination therapy, wherein multiple pharmacological agents are concurrently administered to target different steps of the PG synthesis pathway. The rationale behind combination therapy lies in the hypothesis that synergistic or complementary effects of multiple agents may enhance the likelihood of achieving ductal closure, particularly in cases refractory to monotherapy. While some studies have reported promising results with combination regimens, notably the concomitant use of NSAIDs and acetaminophen, the existing evidence remains limited and heterogeneous. Challenges in interpreting the available data include variations in drug dosing regimens, timing of administration, patient selection criteria, type of use (primary vs rescue), and outcome measures across studies. Furthermore, despite some concerns regarding potential adverse effects, the concomitant use of acetaminophen and NSAIDs seems to be feasible and safe. In clinical practice, the decision to use combination therapy for PDA closure involves careful consideration of the potential risks and benefits, weighing the need for ductal closure against the risk of adverse effects associated with pharmacological treatment. Future research efforts should prioritize well-designed RCTs comparing monotherapy and combination therapy, incorporating standardized protocols, rigorous monitoring of safety outcomes, and long-term follow-up assessments to elucidate the optimal approach for PDA management in premature infants and mitigate associated risks. In addition, exploring a risk stratification-based approach, incorporating more significant clinical markers along with echocardiographic markers, to justify combination therapy.

## Conclusion

Although the combination of acetaminophen and NSAIDs seems to be feasible and safe, it is still uncertain whether the effectiveness of such a management strategy is better than that of either drug alone in the closure of PDA. Therefore, combination pharmacological therapy cannot be recommended for routine PDA closure in preterm infants based on the available evidence. This review highlights the need for a large-scale RCT with an appropriate methodological design to examine the efficacy of combination medical therapy compared to monotherapy on ductal closure and safety assurance.
